# “Reverse life”: A rare case report of situs inversus totalis combined with cardiac abnormalities in a young stroke

**DOI:** 10.1111/cns.13879

**Published:** 2022-06-15

**Authors:** Xue‐qin Chen, Song‐jun Lin, Jian‐jun Wang, Sha Long, Fan‐xin Kong, Zhou‐ke Guo

**Affiliations:** ^1^ Fourth Clinical Medical College of Guangzhou University of Traditional Chinese Medicine Shenzhen China; ^2^ Department of Encephalopathy and Phychology Shenzhen Traditional Chinese Medicine Hospital Shenzhen Guangdong China; ^3^ Department of Ultrasound, Shenzhen Traditional Chinese Medicine Hospital Shenzhen Guangdong China

Dear Editor,

Situs inversus totalis (SIT) is a rare congenital malposition marked by a symmetrical “mirror‐image” arrangement of all internal organs relative to the midline. The estimated incidence of SIT is 1/10,000–1/20,000, of which 2% to 5% are associated with cardiac abnormalities.^1^ Stroke in young age has gained more attention with multiple etiologies, of which cardiac abnormalities are highly correlated with it.^2^ However, stroke in patients with SIT has been rarely reported. Here, we describe a case of a young stroke victim with SIT involving atrial septal defect and ventricular septal defect, which prompts a series of reflective considerations.

A 36‐year‐old woman was admitted to our emergency department for a sudden onset of dysarthria and right extremity weakness for 3.5 h. She had a cesarean delivery 3 months ago and denied any history of hypertension, diabetes, blood transfusion, smoking, or alcohol consumption. Her National Institutes of Health Stroke Scale (NIHSS) was 13 on admission with no aphasia. Neurological examination indicated dysarthria and paresis of right extremities, including upper extremity weakness graded 2/6, while the lower graded 4/6 on the Medical Research Council scale. Urgent computed tomography (CT) of the brain discovered no obvious abnormality(Figure 1A). An emergency cerebral magnetic resonance imaging (MRI) revealed an acute ischemic stroke in the left basal ganglia region (Figure 1B–D).

**FIGURE 1:**
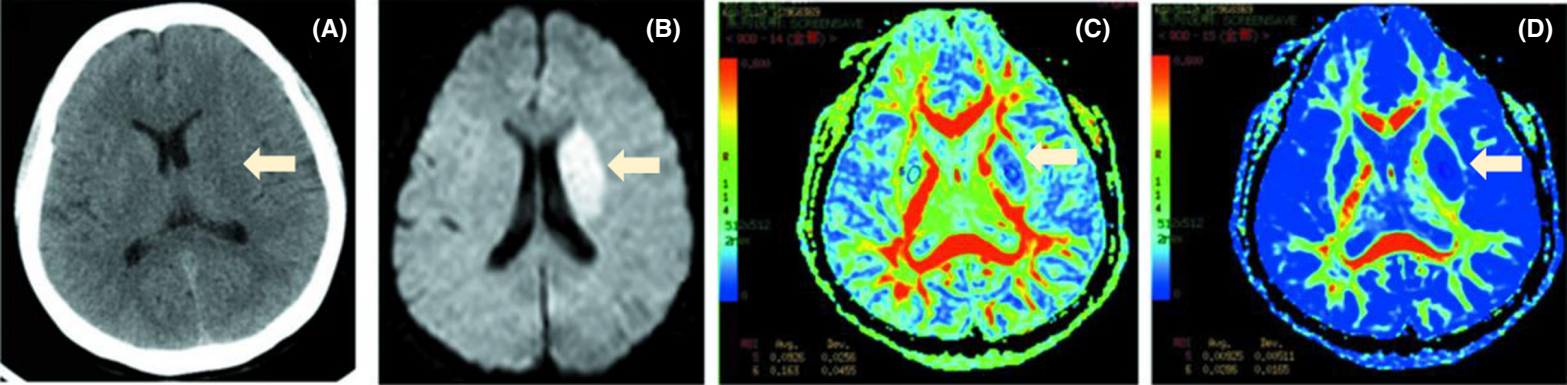
(A) Computed tomography of the brain. (B) Brain magnetic resonance imaging showed an acute ischemic stroke in the left basal ganglia region. (C, D) Diffusion tensor imaging

The patient was treated with Alteplase immediately. Two days later, her NIHSS dropped to 2. Ultrasonography of cervical vessels discovered no occlusions or stenoses of the bilateral carotid and the vertebral arteries of the cervical segment. Laboratory tests, including blood tests, coagulation function test, serum biochemical parameters, tests of kidney and liver function, antibody tests (Syphilis, HIV, anti‐cardiolipin, and autoimmune vasculitis), and rheumatic immunity‐related indicators, were all within normal limits.

Unexpected finding stepped by further investigation. On cardiovascular examination, her apex was heard in right 5th intercostal space 0.5 cm lateral to the midclavicular line, with grade 3/6 ejection systolic murmur. A 12‐lead electrocardiogram yielded results that were consistent with dextrocardia and sinus bradycardia. Ultrasonic cardiogram revealed complex congenital heart disease, including mesocardiac, corrected transposition of the great arteries, and ventricular septal defect (Figure 2A,B). Computed tomography cardiac angiography (CTCA) revealed SIT with dextrocardia, atrial septal defect, interventricular septal defect, and slightly hydrops pericardia. In addition, the whole heart was enlarged and the arteria pulmonalis were widened slightly, and congenital deletion of the left coronary cycloclade was considered (Figure 2D,E). It was consistent with the chest, abdomen, and cavity of pelvis CT and three‐dimensional image (Figure 2C).

**FIGURE 2:**
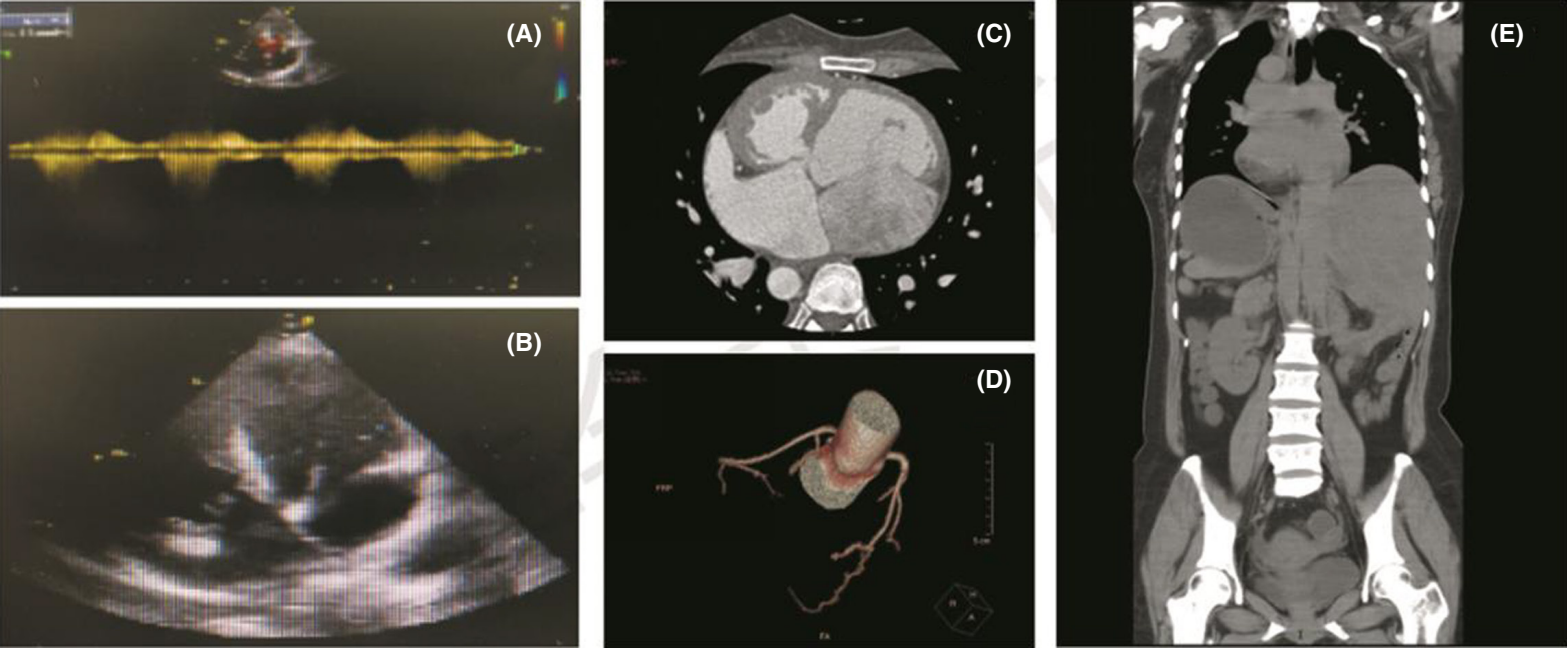
(A, B) Ultrasonic cardiogram. (C, D) Computed tomography cardiac angiography. (E) Chest, abdomen, and cavity of pelvis CT and three‐dimension image suggest total situs inversus, in which the stomach is located on the right side and the liver on the left

The patient was started to be treated with oral antiplatelet aggregation with acenterine, and intravenous drip of butylphthalide. However, due to the patient's financial problems, the examination had to be discontinued. At the time of being discharged from the hospital, she recovered well from previous clinical symptoms with an NIHSS score 0, except with subtle deficits of fine finger movements and positive Babinski's sign on the right side. The patient stopped taking the prescribed drugs Aspirin, Edaravone, and Atorvastatin on her own after one month. During the follow‐up period, she had been free from cerebrovascular episodes and other cardiac complications for the last 4 years.

Situs inversus totalis is a rare abnormality with uncertain pathophysiology. Genetic heterogeneity has proved critical in the variable manifestations,^3^ and more researches have confirmed the association between cilia‐related genes and heterotaxy.^4^, ^5^ Most SIT patients are diagnosed incidentally, for presenting with nonspecific symptoms and unrelated conditions. Strokes in young people with SIT are not commonly reported, and the management of this rare group poses a challenge to clinicians. For them, it is critical to identify the etiology, such as structural cardiac abnormalities, which plays an important role in effective secondary prophylaxis. Therefore, for stroke patients, comprehensive cardiac work‐ups were essential. As the case in our patient, ultrasound and coronary CT confirmed complex precordial disease, in which she had an atrial septal defect and large arterial torsion. Besides, repeated ECGs did not discover risk factors such as atrial fibrillation or arrhythmias.

The term situs and its associated modifiers are used to refer to the entire organism's left–right anatomy. Organ positioning along the left–right axis can be classified into 3 basic classes: situs solitus, referring to the normal left–right anatomical arrangement; SIT, referring to mirror‐image reversal of all organs; and heterotaxy, also known as situs ambiguous, referring to any malposition of organs differing from the first two.^3^, ^6^ Positional abnormalities are described using the terms right, left, and midline, such as in “left‐sided liver.” However, it may cause confusion when describing a structure that is already modified in orientation. For example, shall we rename the patient's structures according to their anatomical location, such as describing the patient's two right‐sided chambers as his right atrium and right ventricle, or defining them as the left atrium and left ventricle located on the right side? Likewise, it is worthwhile to consider how to label the cranial trunk, internal carotid, and subclavian arteries that emanate from the heart of SIT patients.

Classical neuroanatomy, neurophysiology, and neuropathophysiology suggest the brain and limbs have a left–right crossed lateral relationship, e.g., the right side of the brain innervates the strength of left limbs, the left side of the brain senses pain in the right limbs, and the dominant hemisphere in patients with right‐handedness is mostly the left side. Therefore, what would be the lateral relationship between the brain and the limbs of a person with SIT? A noninvasive investigation showed that the frontal and occipital petalia were reversed in individuals with SI.^7^ However, our patient, in accord with a case reported in 1993,^8^ revealed that the classic “left–right crossover” still exists in SIT individuals, in which a left hemispheric stroke caused aphasia and right hemiplegia. Besides, a number of studies have confirmed that the potential mechanisms of visceral organ asymmetries are not necessarily linked to the functional asymmetry of language and handedness.^3^, ^7^, ^9^ Will the “brain network” distinguish between the classical “left–right crossover” to explain the relationship between brain and limb laterality? More supportive research are required to carry out.

## CONFLICT OF INTEREST

The authors declare no conflict of interest.

## CONSENT TO PARTICIPATE

The patient provided written consent for participation.

## CONSENT FOR PUBLICATION

The patient provided written consent for the disclosure of medical information and images.

## Data Availability

All the data and materials have been presented in the main paper.
